# Correspondence on “The Open DAC 2023 Dataset and Challenges for Sorbent Discovery in Direct Air Capture”

**DOI:** 10.1021/acscentsci.5c00255

**Published:** 2025-05-29

**Authors:** Xin Jin, Susana Garcia, Berend Smit

**Affiliations:** † Laboratory of Molecular Simulation (LSMO), Institut des Sciences et Ingénierie Chimiques, 27218 École Polytechnique Fédérale de Lausanne (EPFL), Rue de l’Industrie 17, CH-1951 Sion, Switzerland; ‡ The Research Centre for Carbon Solutions (RCCS), School of Engineering and Physical Sciences, 3120 Heriot-Watt University, EH14 4AS Edinburgh, United Kingdom

## Abstract

This Correspondence provides a brief commentary on a recent *ACS Central Science* article that identifies metal–organic frameworks for direct air capture and suggests that the recommended structures are artifacts of the methodology used.

## Graphical abstract

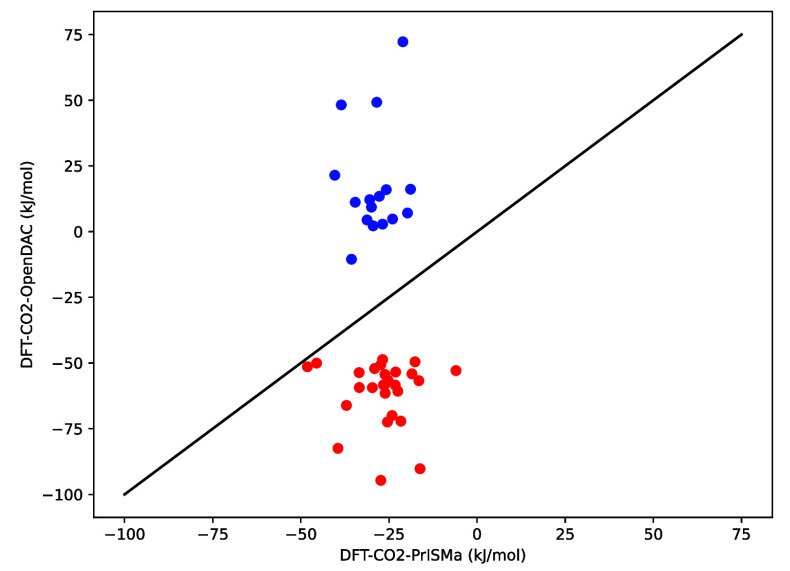

The recent article Sriram et al.^1^ carried out a detailed screening of metal–organic frameworks (MOFs) for direct air capture (DAC). In this work, a promising material was defined as one with a binding energy of CO_2_ that is sufficiently large (i.e., $-E_{bind}^{{CO}_{2}}\;>$ 50 kJ mol^–1^) and larger than the corresponding binding energy of water $\left(-E_{bind}^{H_{2}O}<-E_{bind}^{{CO}_{2}}\right)$ . The OpenDAC project identified 135 MOF structures, out of 5961 pristine MOF structures, that met this criterion. These results are important as one could envision a DAC process using physisorbents instead of amine-based chemisorbents. As these amines oxidize with the oxygen in the air, having a process with materials that do not deteriorate would be a significant step forward. There are many reasons why the OpenDAC 2023 study has attracted the scientific community’s attention. The large size of the data set and its open-source availability make this study a valuable resource for the community. In addition, the study focuses on two key challenges often ignored in screening studies for carbon capture applications. First, all CO_2_ sources contain water, and describing the interactions of water with metal–organic frameworks using classical force fields is extremely challenging. Second, most screening studies, if not all, assume MOF crystals to be rigid. The OpenDAC 2023 is one of the first large-scale computations considering flexible MOFs. In this correspondence, we report on our efforts to reproduce the OpenDAC’s binding energy calculation. In Figure 1, we compare our calculations with those of the OpenDAC project. Our calculation (which we refer to as PrISMa) uses the same materials from the CoReMOF 2019 database.^2^ However, we could find only one MOF (ASEJOZ01) that meets the requirements for DAC (see Figure 2). This structure was not in OpenDAC’s list of promising MOFs. There are various reasons for these differences.

**Figure 1:**
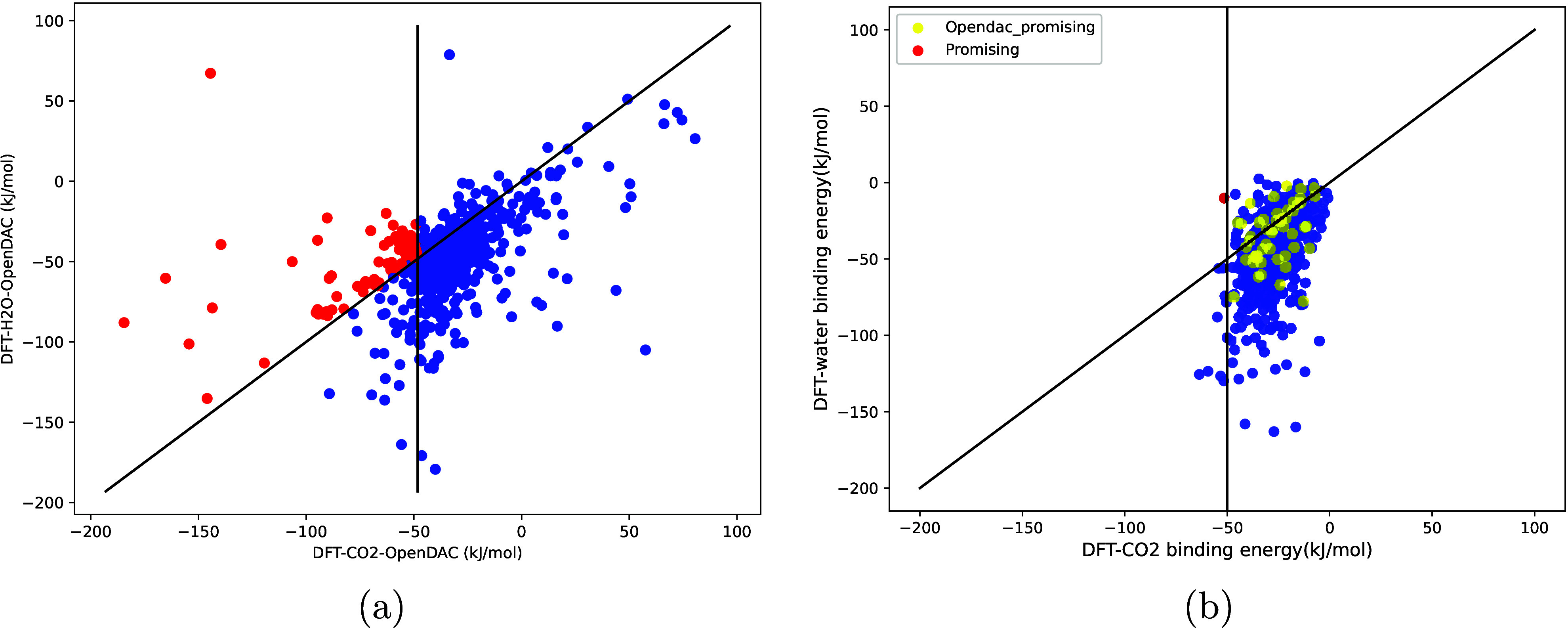
Water binding energy versus carbon dioxide binding energy (a) as obtained by OpenDAC^1^ and (b) this work. The red dots are MOFs that meet the criteria of being promising for DAC (i.e., $-E_{bind}^{{CO}_{2}}\;>$ 50 kJ mol^–1^ and $-E_{bind}^{H_{2}O}<E_{bind}^{{CO}_{2}}$ ).

**Figure 2:**
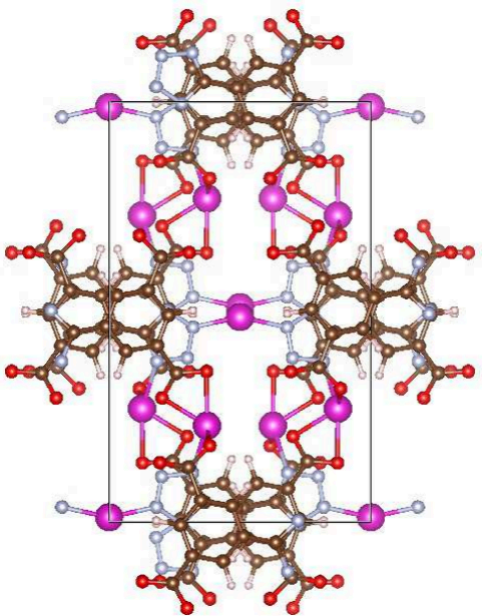
MOF structure ASEJOZ01.

Before computing the binding energies, we removed those structures from the OpenDAC database, which correspond to structures from the CoRE MOF database that are flagged by the MOFchecker^3^ as being “charged”. Each MOF structure name in the CCDC database corresponds to a specific experimentally reported framework. Hence, by definition, the original experimental structure must have been neutral. However, some MOF structures reported in the CoRE MOF database have a net charge because they have missing hydrogen atoms or have been cleaned incorrectly (e.g., solvent molecules that have been removed carry a net charge). These “charged” structures can often pass the DFT optimization. If it passes, one obtains converged neutral structures. We discard these because they do not represent the experimental structures the calculations intended to model.

About 40% of the underlying CoRE MOF structures used in the OpenDAC data set were flagged as being “charged”. This is to be expected, as many structures in the OpenDAC data set originate from the 2019 CoRE MOF database. Zhao et al.^4^ also pointed out that the earlier versions of the CoRE MOF database (CoRE MOF 2014 and 2019)^2,5^ contained some incorrect structures. In the updated version (CoRE MOF 2025), the MOFchecker was employed as one of the validation tools. These discarded structures gave unrealistically high binding energies.

The SI lists the recommended structures we discarded from the OpenDAC project. The full list of problematic structures can be found on Zenodo.^6^

Because of limited computational resources, we only compared MOF structures with a closed-shell configuration. Additionally, the number of atoms in the unit cell is restricted to fewer than 500. To avoid additional interactions between adsorbate molecules, the unit cell must be sufficiently large to ensure that the intermolecular distance between adsorbates exceeds 8 Å.

The DFT calculations of Sriram et al.^1^ were carried out using VASP with PBE-D3. Our calculations were done with the same functional (PBE) and van der Waals correction (D3), but using CP2K.^7^ The Gaussian basis sets were double-ζ with one set of polarization functions (DZVP), and the pseudopotentials we applied are the Goedecker–Teter–Hutter (GTH) pseudopotentials. We compared a set of structures with some single-point energies and found good agreement between the DFT results of the OpenDAC project and the DFT calculations presented here.

Many groups have used variations of the method used by Sriram et al.^1^ to optimize structures from the CoRe-MOF 2019 database. For example, to compute the mechanical properties^8^ or the heat capacity^8^ of MOFs, the optimized structure is the reference point of the calculation. In addition, many groups use DDEC charges to compute adsorption isotherms, and these DDEC charges are computed from the optimized structure.^9^

The above examples are from our group, but many other studies published in the literature use a similar optimization technique as in the OpenDAC project. There is, however, an important difference. If we compute the mechanical properties or heat capacity, we make a small perturbation of the optimized structure. The results are reasonable as long as the optimized structure is in a local minimum that is reasonably close to the true minimum.

The calculation of the energy minimum is fundamentally different, as we compute the energy difference between two configurations: the MOF with a guest molecule and the pristine MOF. To get the binding energy, we need to subtract two large numbers. However, adding a guest molecule is not a small perturbation and can cause the MOF to be kicked out of the local minimum found in optimizing the pristine MOF.

It is important to note that Sriram et al.^1^ did mention the potential problems and pitfalls of the CoReMOF 2019 database and the possibility that the inclusion of adsorbate molecules during relaxation broke framework symmetry and resulted in lower energy empty MOF configurations.

To obtain the binding energies, the OpenDAC project computes the difference between the energy-minimized structure of a MOF structure with a water or CO_2_ molecule and the pristine MOF. Our workflow differs on an essential point. After minimizing the MOFs with either a water or CO_2_ molecule, we remove the water or CO_2_ molecule and energy-minimize the empty structures. This approach gave us three empty MOFs for which we have three different minimum energies: the pristine MOF, the MOF with the H_2_O molecule removed, and the MOF with the CO_2_ removed. We used the value corresponding to the one with the lowest energy to compute the binding energy.

Interestingly, we could not find any systematics in which one of the three energies would be minimal. At this point, it is important to note that although our method gives more reliable binding energy estimates, we are not guaranteed to obtain the absolute minimum energy configurations. By minimizing after removing a CO_2_ or H_2_O molecule, we “kick” the system out of a local minimum, and by selecting the minimum, we are guaranteed to have a better estimate. However, if we include calculations of the binding energy of, say, H_2_S, we probably find an even lower energy of the empty MOF for some MOFs. In Section S1 of the SI, we give a more detailed discussion of these results.

In Figure 3, we compare our binding energies for CO_2_ with those of the OpenDAC project for a set of structures. Our binding energies are in the range −50 to −10 kJ mol^–1^ while OpenDAC reports binding energies −100 to 75 kJ mol^–1^. Experimentally, one can compare the binding energy with the heat of adsorption, which are typically in the range −50 to −10 kJ mol^–1^. The very low and high values of the binding energies found by the OpenDAC project are artifacts. They are caused by using local minima to compute the binding energy. Consequently, the machine learning models provided to predict the binding energies should be retrained with the correct data.

**Figure 3:**
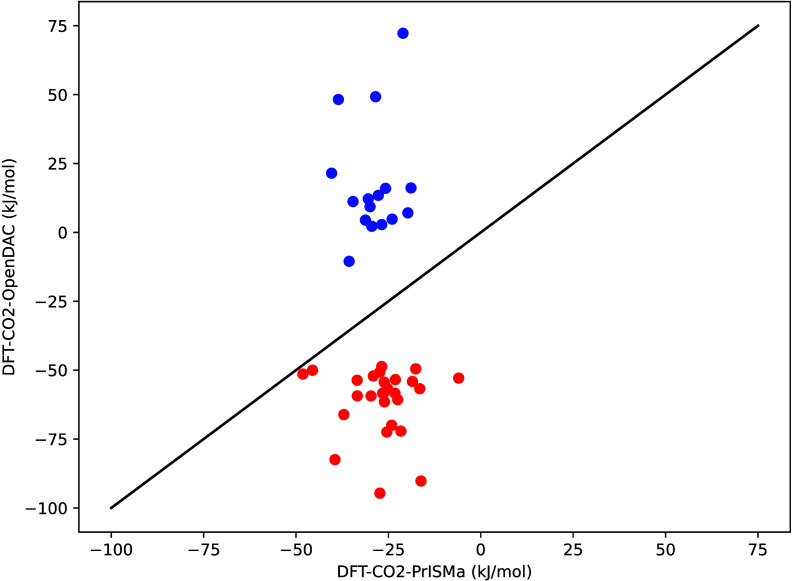
Comparison of the CO_2_ binding energy from our DFT calculations with the DFT calculations of OpenDAC.^1^ The blue dots are structures for which OpenDAC reported a positive CO_2_ binding energy, and the red ones are those with a reported binding energy lower than −50 kJ mol^–1^. The full list can be found in Table S2 of the SI.

Also, we would like to emphasize that in this Correspondence, we narrowly focused on the binding energy, which is just one aspect of the OpenDAC project. The use of DFT relaxation in the OpenDAC work takes an important step beyond the more typical use of rigid structures in screening calculations.^7^ As emphasized by Sriram et al.,^1^ these relaxations can have an important impact on the adsorption properties and should be a routine check in the workflow of these screening studies. The OpenDAC project also reports large amounts of DFT results. The method used to compute the binding energies does not impact these DFT results. However, if the structures taken from the CoRE MOF database are charged or problematic in other ways, these results should be used with care. In Table S1 of the SI, we have flagged all problematic structures in the list of promising ones for DAC. The list of all problematic structures can be found on Zenodo.^6^

Finally, we want to emphasize that this Correspondence was made possible because the OpenDAC project made all its data publicly available.

## Supplementary Material



## Data Availability

The full list of problematic structures, as well as the numerical data of Figure 1b, are available on Zenodo (https://zenodo.org/records/14802658).^6^

## Notes

Binding energy calculation details, a list of problematic MOF structures, and the numerical data of Figure 3 (PDF)
