# Supplementary material for: Correspondence on “The Open DAC 2023 Dataset and Challenges for Sorbent Discovery in Direct Air Capture”
Source: ACS Cent Sci. 2025 May 29;11(6):868–71. doi: 10.1021/acscentsci.5c00255 (PMC12203258; doi:10.1021/acscentsci.5c00255)
Supplement: Supplementary file 1 [file oc5c00255_si_001.pdf]

Supporting Information: Correspondence on "The Open DAC 2023 Dataset and Challenges for Sorbent Discovery in Direct Air Capture"

Xin Jin,[†] Susana Garcia,[‡] and Berend Smit*,[†]

†*Laboratory of Molecular Simulation (LSMO), Institut des Sciences et Ingénierie Chimiques, École Polytechnique Fédérale de Lausanne (EPFL), Rue de l'Industrie 17, CH-1951 Sion, Switzerland*

‡*The Research Centre for Carbon Solutions (RCCS), School of Engineering and Physical Sciences, Heriot-Watt University, EH14 4AS Edinburgh, United Kingdom*

E-mail: berend.smit@epfl.ch

Contents

1 Energy minimization **S-3**

1.1 Pristine MOFs S-3

1.2 Binding energy calculation S-7

1.3 Data sets S-7

2 List of problematic structures **S-9**

3 Comparison of binding energies of selected structures **S-13**

References **S-15**

1 Energy minimization

1.1 Pristine MOFs

We computed, for a subset of 712 MOFs used in the OpenDAC project, the following three (total) energies:

- E_{Pristine}, which is the energy after optimizing the pristine MOF,
- $E_{\text{MOF}_{\text{H}_2\text{O}}}$, which is the energy after removing a water molecule from the optimized configuration with a H_2O molecule and subsequent optimization. The starting point for this calculation was the optimized structure provided by the OpenDAC project.
- $E_{\text{MOF}_{\text{CO}_2}}$ which is the energy after removing a CO_2 molecule from the optimized configuration with a CO_2 molecule and subsequent optimization. Also, for this calculation, we used the optimized structure provided by the OpenDAC project as our starting point.

In Figure S1, we summarize our observation of these different energies.

Figure S1a shows the ratios of $E_{\text{MOF}_{\text{H}_2\text{O}}}/E_{\text{Pristine}}$ (blue) and $E_{\text{MOF}_{\text{CO}_2}}/E_{\text{Pristine}}$ (red). For MOFs that have a ratio > 1, E_{Pristine} is *not* the lowest energy.

The data in Figure S1a suggest that if we look at the absolute energies, we observe that the differences between these three energies are small. However, as is shown in Figure S1b these tiny differences ($\ll 1\%$) have large effects on the binding energy. We observe differences as large as $100\,\text{kJ}\,\text{mol}^{-1}$, which is, given that the average binding energy is $-60\,\text{kJ}\,\text{mol}^{-1}$ to $-50\,\text{kJ}\,\text{mol}^{-1}$, a very significant error.

In Figure S1b, we have ordered the MOFs in terms of absolute differences between the computed energies. This ranking is different for the different energies. If we focus on $E_{\text{MOF}_{\text{H}_2\text{O}}} - E_{\text{Pristine}}$ (blue), we see three regimes. There are about 40 structures for which the $E_{\text{Pristine}} > E_{\text{MOF}_{\text{H}_2\text{O}}}$, for which we observer negative values in Figure S1b. Using E_{Pristine}

will overestimate the binding energy for these structures. Interestingly, there are also structures for which $E_{\text{Pristine}} < E_{\text{MOF}_{H_2O}}$. Adding a water molecule into these structures puts the MOF at a local minimum with a higher energy. For $E_{\text{MOF}_{CO_2}} - E_{\text{Pristine}}$ (red) we see a similar trend.

It is also interesting to look at $E_{\text{MOF}_{CO_2}} - E_{\text{MOF}_{H_2O}}$ (green). One could argue that it is important to perturb the system to get it out of a local minimum, and it should not matter whether we achieve this with a H_2O or CO_2 molecule. If this were the case, the green dots would be all zero, as $E_{\text{MOF}_{CO_2}} \approx E_{\text{MOF}_{H_2O}}$. That we see both positive and negative differences indicates that it is rather random whether CO_2 of H_2O gives us the lowest energy. This is also the conclusion that can be drawn from Figure S1c. This figure indicates which of the three energies is the lowest, the middle, and the highest for each MOF. Of the 15% of MOFs for which the pristine MOF does not give the lowest energy, for about 50%, the lowest energy is obtained with H_2O and the rest with CO_2.

These conclusions should also raise a red flag. Suppose that we were to include a third guest molecule in our calculations (e.g., H_2S, NOx, SOx, etc.), then we will most likely find MOFs for which this third guest will give the lowest energy. Hence, we can only conclude that our binding energies are a better estimate than the OpenDAC project. However, there is *no* guarantee that our results would not improve with the inclusion of additional guest molecules.

For instance, we developed a scheme to optimize COFs in which, during the optimization step, we carry out some high-temperature ab initio MD to push the system out of such local minima.[S1] We often achieve a lower energy than the initial minimization step. This initial step was similar to the one used by the OpenDAC project. However, this is a relatively expensive procedure. Interestingly, minimizing with a guest molecule can also push the system out of local minima and is a much cheaper way of achieving this. We also want to emphasize that this ab initio MD method, like any other method, does not guarantee that an absolute minimum will be found.

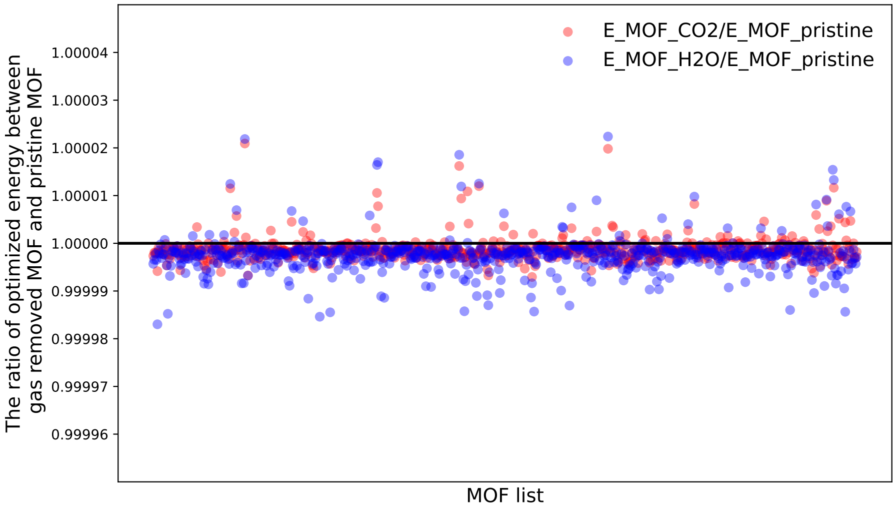
E_MOF_CO2/E_MOF_pristine
E_MOF_H2O/E_MOF_pristine
1.00004
1.00003
1.00002
1.00001
1.00000
0.99999
0.99998
0.99997
0.99996
The ratio of optimized energy between gas removed MOF and pristine MOF
MOF list

(a)

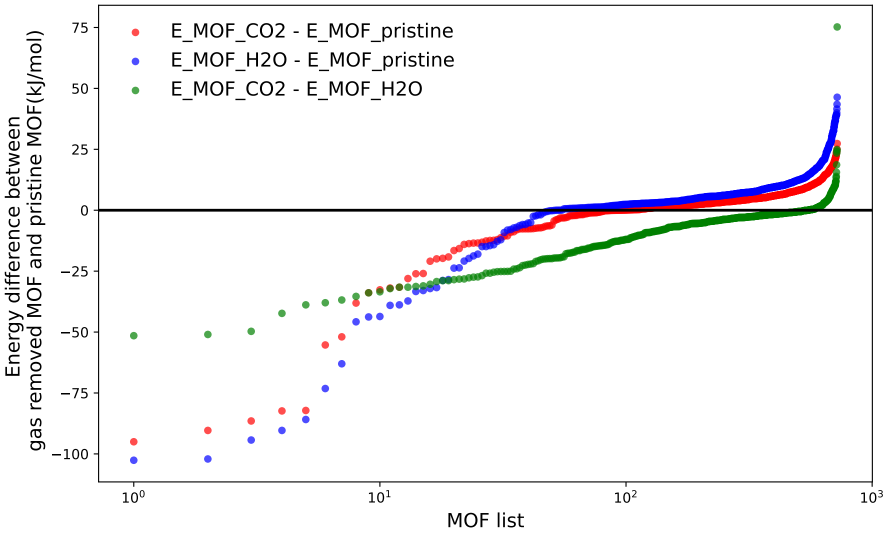

(b)

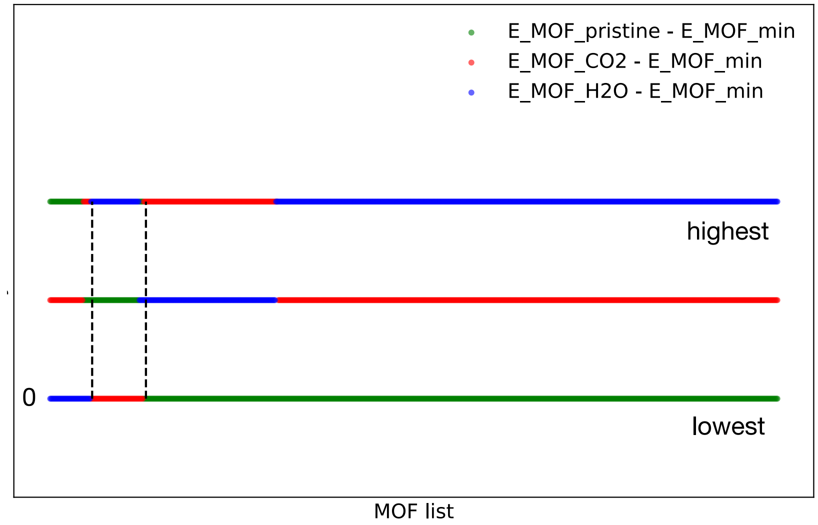

(c)

Figure S1: Minimum energies for MOFs optimized with the pristine structure E_{Pristine}, optimized after removing a water molecule $E_{\mathrm{MOF}_{\mathrm{H_2O}}}$, and optimized after removing a CO_2 molecule $E_{\mathrm{MOF}_{\mathrm{CO_2}}}$. (a) gives the ratios of $E_{\mathrm{MOF}_{\mathrm{H_2O}}}/E_{\mathrm{Pristine}}$ (blue) and $E_{\mathrm{MOF}_{\mathrm{CO_2}}}/E_{\mathrm{Pristine}}$ (red) for a MOF. (b) gives the impact of using a different reference for the empty MOF in $\mathrm{kJ\,mol^{-1}}$. We have ordered the list of MOFs using these differences in this figure. (c) shows for each MOF which energy is the lowest (bottom line), middle (middle line), and highest (top line) $E_{\mathrm{MOF\_min}}$ is the minimum of the three energies. The lowest is, by definition, zero. The other differences (middle and highest) are scaled for each MOF to give the same values. In these figures, the horizontal axis is a list of MOFs. In Figures (a) and (c), a point on the horizontal axis represents the same MOF, while in Figure (b), the list is ordered in increasing energy difference. Hence, a point on the horizontal axis represents a different MOF for the three energy differences. All data can be found on Zenodo.[S2]

1.2 Binding energy calculation

In our calculation of the binding energies of CO_2 and H_2O, we did not recompute the minimum energy configuration of the MOF with a CO_2 or H_2O as obtained by the OpenDAC project. The positions of the adsorbates are taken from the ODAC23 dataset to enable comparison with the corresponding binding energy results. For the most promising structures, we optimized the structures with CO_2 and H_2O molecules independently. We took the structure with the lowest energy, i.e., the one obtained by the OpenDAC project or ours (see Table S2).

A surprising observation is that our calculations indicate that 15% of the structures have an incorrect binding energy. In the OpenDAC project, there are 135 recommended structures, of which about 71 are based on structures from the CoRE-MOF database that were charged MOFs. If we discard those, we would still expect about 64 MOFs, potential candidates for DAC. We found none, which illustrates the unfortunate fact that top performers resulted from an incorrect estimate of the binding energy. Therefore, one can expect very few MOFs with the desired properties.

1.3 Data sets

We uploaded on Zenodo[S2] three files with the results of the energy calculations. The unit of the binding energy is $\mathrm{kJ\,mol^{-1}}$, and the units of the rest of the data are a.u.

- `water_binding_energy.csv`, which contains the bsse corrected energy of MOF with H_2O, minimum pristine MOF energy, energy of H_2O, and the binding energy of H_2O.
- `CO2_binding_energy.csv`,which contains the bsse corrected energy of MOF with CO_2, minimum pristine MOF energy, energy of CO_2, and the binding energy of CO_2.
- `mof_energy.csv`, which includes the MOF energy of the pristine MOF structures and the empty optimized MOF structures obtained after removing CO_2 or H_2O.

The file `mof_energy.csv` gives the energy differences between these three different optimized structures. These differences demonstrate that calculating only the optimized pristine MOF structures may affect the final binding energy.

2 List of problematic structures

In Table S1, we list all promising structures from the OpenDAC project.[S3] Those for which we detected a problem, the Qualification "wrong" is given together with a short description. We used the `MOFchecker` to detect these problems with the structures.[S4] The complete list of structures can be found on Zenodo https://zenodo.org/records/14802658.[S2]

Table S1: Analysis of the CIF files for the most promising structures from the OpenDAC project.[S3] These structures were shortlisted based on the CO_2 and H_2O DFT binding energy values: (1) the CO_2 binding energy $< -0.5\,\mathrm{eV}$ and (2) the CO_2 binding energy $>$ the H_2O binding energy, in absolute values. The structure label refers to the Cambridge Crystallographic Data Centre (CCDC) identifier. Structures can be visualized using our Structure Visualization Tool

Label	Qualification	Description	Article (DOI)
ADOCEC	wrong	charged	10.1016/j.poly.2006.11.050
ANUGOG	right	-	10.1021/ja107423k
BAXFUD	wrong	charged	10.1021/ic202626c
BEXQIF	wrong	charged	10.1021/cg034258n
BIMDIL	right	-	10.1021/ja003159k
BOWNUY	wrong	missing O, charged	10.1039/C4RA16350H
CAVNOE	wrong	missing H, charged	10.1039/c1dt11729g
CAVSUP	wrong	missing H, charged	10.1039/c2dt11992g
cg901128k_si_004	wrong	charged	10.1021/cg901128k
cm501138g_si_002	right	-	10.1021/cm501138g
cm503311x_alf175k	wrong	missing H, charged	10.1021/cm503311x
cm503311x_alf350k	wrong	missing H, charged	10.1021/cm503311x
cm503311x_alf400k	wrong	missing H, charged	10.1021/cm503311x
cm503311x_alf425k	wrong	missing H, charged	10.1021/cm503311x
cm503311x_aloh225k	wrong	missing H, charged	10.1021/cm503311x
cm503311x_aloh500k	wrong	missing H, charged	10.1021/cm503311x
CUQXUI	wrong	charged	10.1016/j.solidstatesciences.2010.07.004
DEBWAK	right	-	10.1002/anie.201200758
DEJRUH	wrong	charged	10.1002/chem.201202377
DICKEH	right	-	10.1021/ic302018g
DITYOW	wrong	charged	10.1039/C3NJ01198D
ECEZAO01	wrong	charged	10.1021/cg050363g
EGEJIK_manual	right	-	10.1002/anie.200802908
EGIFUV	wrong	charged	10.1021/ja020480p
ESUSOC01	right	-	10.1021/jacs.5b09231
ESUVAR01	right	-	10.1021/jacs.5b09231
EVEGOB	wrong	missing H, charged, under coord. carbon	10.1021/ja039472e

Table S1: (continued)

Label	Qualification	Description	Article
EXALAR	wrong	charged	10.1021/cg200261j
FAGQAI	right	-	10.1002/ejic.201501194
FECXES	right	-	10.1039/c2jm15538a
FEWGUL	right	-	10.1021/ic302318j
FEYJOJ	right	-	10.1021/ic048612y
FIZPOV	right	-	10.1021/cg401887b
FUNLAD	right	-	10.1038/ncomms8954
FUVDEH04	wrong	over coord. carbon	10.1021/jacs.5b03280
FUVDEH05	wrong	over coord. carbon	10.1021/jacs.5b03280
FUVDEH06	wrong	over coord. carbon	10.1021/jacs.5b03280
FUVFUZ	wrong	over coord. carbon	10.1021/jacs.5b03280
FUVGAG02	wrong	over coord. carbon	10.1021/jacs.5b03280
GIJXAA	wrong	replicated linker	10.7868/S0132344X13020011
GOSBOH	wrong	charged	10.1016/j.poly.2014.09.039
GUHNUU	right	-	10.1021/cm503533r
HOMRIL	wrong	charged	10.1021/ic801338b
HUFJEZ	wrong	charged	10.1002/ejic.201500647
HUQHOS	right	-	10.1002/chem.201502758
IBUDOZ	right	-	10.1002/ejic.200400170
IFEHUY	right	-	10.1021/ja4037516
IPIDUH	right	-	10.1016/j.inoche.2010.10.015
IPIFAP	wrong	charged	10.1016/j.inoche.2010.10.015
IXEJOM	right	-	10.1002/anie.201604313
IYEHAX	wrong	missing H, charged	10.1039/C6QI00273K
JOCWIJ	wrong	charged	10.1021/ic403148f
jp302979a_si_002	wrong	replicated O	10.1021/jp302979a
KEDJAG16	wrong	charged	10.1039/c2dt30818e
KEHGAH_charged	wrong	charged	10.1039/c2dt30362k
KEHGIP	wrong	charged	10.1039/c2dt30362k
KIPKEB	right	-	10.1021/cg400606z
KIPZEQ	wrong	charged	10.1021/cg4002626
KOQLUZ	wrong	charged	10.1039/C4CE01725K
LAZJIH	wrong	charged	10.1021/cg201362x
LEWZET	wrong	missing H, charged	10.1002/anie.200604164
LOXWAY	right	-	10.1002/anie.201408933
LUDKOM	wrong	replicated linker	10.1039/c3nr04161a
LUFQUZ	right	-	10.1021/ja902187d
MALRUM	wrong	charged	10.1016/S0020-1693(99)00380-1
MIXBIG_auto	right	-	10.1039/C3CC49457H
MOFKUO	wrong	charged	10.1021/ic7023082
NASREH	right	-	10.1021/acs.cgd.7b00118

Table S1: (continued)

Label	Qualification	Description	Article
NAYHAY	wrong	charged	10.1039/c2dt30078h
NEDWEA	right	-	10.1021/ja303092m
NEJSOM	right	-	10.1126/science.1231451
NETYIV	wrong	charged	10.1002/ejic.200600558
NEVVEQ	right	-	10.1002/anie.200601627
NUBBAP	wrong	charged	10.1039/C4CE02154A
ODIXEG	right	-	10.1039/C3CE41105B
OLOPEM	right	-	10.1039/C6QI00067C
OPIYAP	wrong	free water	10.1016/j.micromeso.2016.06.013
OSUWEF	wrong	charged	10.1080/15533174.2011.568430
PANYOU_charged	right	-	10.1021/cg201181s
PETWIW	wrong	replicated linker	10.1039/c3cc37695h
PIJJOI	right	-	10.1021/cg070320v
POMSOB	right	-	10.1016/j.inoche.2014.07.015
PUGGOP	right	-	10.1021/ic5018517
QAWPIO	wrong	charged	10.1039/b508930a
QEFNAQ	right	-	10.1021/ja002624a
QERZUI	right	-	10.1021/cg005519l
QERZUI01	right	-	10.1039/b200213b
QODHUO03	wrong	charged	10.1039/C3DT53580K
QODHUO07	wrong	charged	10.1039/C3DT53580K
QOFDIA02	wrong	charged	10.1039/C3DT53580K
QONKUB	right	-	10.1021/ic5008457
QOVSOL	right	-	10.1039/C4CE02551B
QUQFOY	right	-	10.1021/cg100316s
RIPKIM	right	-	10.1039/C3DT52266K
RUBTAK03_auto	right	-	10.1021/cg501386j
science.1056698_manual	right	-	-
SOBZEQ	right	-	10.1016/j.molstruc.2014.03.027
SUSZOW	wrong	not a MOF	10.1021/cm902528y
TAPLED	wrong	charged	10.1039/c0ce00517g
TEMPEI	right	-	10.1016/j.crci.2012.09.001
TIDLEZ	wrong	charged	10.1039/c2ce06397b
TIDLID	wrong	charged	10.1039/c2ce06397b
TONWUO	wrong	charged	10.1016/0277-5387(96)00127-1
UCOCUM_neutral	right	-	10.1021/ja203695h
UFUNIS	right	-	10.1039/b200213b
UHASED	right	-	10.1021/cg501706t
UTEWUM	right	-	10.1039/c1sc00136a
VAXMUF	wrong	charged	10.1039/C7CE00319F
VEGMOK01_charged	right	-	10.1107/S0108768105042795

Table S1: (continued)

Label	Qualification	Description	Article
VUWSAJ	right	-	10.1039/C5RA10799G
WAHKUO	right	-	10.1039/C5CC10203K
WIHWAN	right	-	10.1016/j.jssc.2012.12.030
WIKLOT	wrong	charged	10.1021/ic400658y
WIRMOB	right	-	10.1039/C3CE41459K
XAFXOT	right	-	10.1039/c001537g
XALDAS	right	-	10.1021/acs.cgd.7b00007
XAMDUM07	right	-	10.1039/c1cc16045a
XANMUX04	wrong	charged	10.1021/acs.cgd.7b00060
XAZGAG_charged	wrong	charged	click here
XEDPON	wrong	charged	10.1039/c2cc32927a
XENCAU	wrong	charged	10.1039/b007183h
XEXMEU	right	-	10.1021/ic302334x
XINWUO	wrong	charged	10.1016/j.inoche.2013.01.023
XITYOP	right	-	10.1021/ja0771639
XOVPUU	wrong	charged	10.1021/ic801631w
XUVHEB	right	-	10.1016/S0022-4596(03)00141-5
YILJAG	right	-	10.1002/anie.201307217
YINXIE	right	-	10.1021/cg400680r
YIWNEX	wrong	missing H, charged	10.1039/dt9940003695
YUGLES	right	-	10.1039/B917046D
ZAFXAI	wrong	charged	10.1002/anie.201506345
ZAGHAT	wrong	charged	10.1021/acs.inorgchem.5b02030
ZIDBEV	wrong	charged	10.1039/C3RA41567H
ZIHFUR	wrong	charged	10.1039/C39950002199
ZILFOR_manual	right	-	10.1039/C3TA12662E

3 Comparison of binding energies of selected structures

From the OpenDAC project, we selected some structures that had an unexpected (unrealistic) binding energy, i.e., MOFs with a positive CO_2 binding energy or with a reported binding energy lower than $-50\,\mathrm{kJ\,mol^{-1}}$.[S3] These results are shown in the column "OpenDAC (DFT)." We compared these results with our PrISMa workflow to compute the binding energy. We compute the binding energy both with the UFF force field ("PrISMa (FF)") as well as with DFT ("PrISMa (DFT)"). The "PrISMa label" refers to the structures reported by Charalambous et al.[S5].

Table S2: Details of the binding energy ($\mathrm{kJ\,mol^{-1}}$) data. The "OpenDAC label" refers to the structures reported by the OpenDAC project[S3] and the "PrISMa label" to structures reported by Charalambous et al.[S5]. The columns compare the DFT calculations of the CO_2 binding energy by the OpenDAC project ("OpenDAC (DFT)") with those computed in this work, in which we used the UFF force field ("PrISMa (FF)") or DFT ("PrISMa (DFT)").

OpenDAC label	PrISMa label	OpenDAC (DFT)	PrISMa (FF)	PrISMa (DFT)	Promising
ATULIM	RSM1647	-10.51	-36.03	-35.63	No
BIMDIL	BIMDIL	-52.89	-27.83	-5.98	Yes
BUQWER		13.44	-35.98	-27.73	No
cm501138g_si_002	cm501138g_si_002	-58.33	-23.24	-26.26	Yes
DEBWAK	DEBWAK	-70.01	-19.48	-24.12	Yes
DICKEH	DICKEH	-54.35	-17.47	-26.14	Yes
DORFOG	RSM0290	15.97	-37.20	-25.76	No
FAGQAI	FAGQAI	-72.13	-14.23	-21.62	Yes
FECXES	FECXES	-61.50	-26.72	-26.08	Yes
FEWGUL	FEWGUL	-50.65	-26.02	-27.31	Yes
FIZPOV	FIZPOV	-72.45	-16.70	-25.43	Yes
FUNLAD	FUNLAD	-49.53	-24.83	-17.60	Yes
GALCAZ	RSM3405	12.18	-24.91	-30.46	No
GERNOI	RSM3849	16.13	-28.27	-18.88	No
GIDKOU	RSM2953	48.23	-40.34	-38.53	No
GUHNUU	GUHNUU	-53.43	-18.88	-23.11	Yes

Table S2: (continued)

OpenDAC label	PrISMa label	OpenDAC (DFT)	PrISMa (FF)	PrISMa (DFT)	Promising
HOWPOZ	RSM2898	11.19	-28.97	-34.57	No
IBUDOZ	IBUDOZ	-51.42	-35.65	-48.13	Yes
KESGAS	RSM2467	4.81	-30.06	-23.98	No
KIPKEB	KIPKEB	-58.38	-23.23	-23.22	Yes
LOXWAY	LOXWAY	-59.33	-23.63	-33.41	Yes
NANMEW	RSM2581	9.32	-36.22	-29.97	No
NEJSOM	NEJSOM	-54.12	-26.55	-18.48	Yes
NUVYIN	RSM3145	21.47	-34.10	-40.37	No
ODIXEG	RSM1197	-90.23	-19.75	-16.18	Yes
OLOPEM	OLOPEM	-60.71	-32.32	-22.48	Yes
OTAVOV	RSM2754	72.26	-23.24	-21.05	No
PIJJOI	PIJJOI	-94.66	-32.70	-27.31	Yes
POHCOG	RSM3812	4.45	-23.90	-31.22	No
POMSOB	POMSOB	-50.03	-31.09	-45.53	Yes
QERZUI	QERZUI	-82.47	-38.42	-39.46	Yes
QERZUI01	QERZUI01	-66.12	-39.56	-37.06	Yes
QONKUB	QONKUB	-56.73	-20.45	-16.51	Yes
SABKIS	RSM2998	2.20	-33.38	-29.49	No
SOBZEQ	SOBZEQ	-59.37	-23.87	-29.70	Yes
TOHYUM	RSM1124	49.24	-27.77	-28.47	No
VUNNEZ	RSM0118	2.84	-24.86	-26.81	No
WALKAY	RSM2771	7.08	-34.41	-19.74	No
WIHWAN	WIHWAN	-53.65	-18.79	-33.46	Yes
XAMDUM07	XAMDUM07	-56.55	-23.40	-25.30	Yes
XEXMEU	XEXMEU	-48.69	-22.90	-26.81	Yes
XUVHEB	XUVHEB	-52.11	-27.36	-29.07	Yes

References

(S1) Ongari, D.; Yakutovich, A. V.; Talirz, L.; Smit, B. Building a consistent and reproducible database for adsorption evaluation in covalent–organic frameworks. *ACS central science* **2019**, *5*, 1663–1675.

(S2) Jin, X. Correspondence on Opendac2023. 2025; https://zenodo.org/records/15118876, Accessed: 2025-04-01.

(S3) Sriram, A.; Choi, S.; Yu, X. H.; Brabson, L. M.; Das, A.; Ulissi, Z.; Uyttendaele, M.; Medford, A. J.; Sholl, D. S. The Open DAC 2023 Dataset and Challenges for Sorbent Discovery in Direct Air Capture. *Acs Central Sci* **2024**, *10*, 923–941, DOI: 10.1021/acscentsci.3c01629.

(S4) Jin, X.; Jablonka, K.; Moubarak, E.; Li, Y.; Smit, B. MOFChecker: An algorithm for Validating and Correcting Metal-Organic Framework (MOF) Structures. *ChemRxiv* **2025**, DOI: 10.26434/chemrxiv-2025-bh607.

(S5) Charalambous, C.; Moubarak, E.; Schilling, J.; Sanchez Fernandez, E.; Wang, J.-Y.; Herraiz, L.; Mcilwaine, F.; Peh, S. B.; Garvin, M.; Jablonka, K. M.; Moosavi, S. M.; Van Herck, J.; Ozturk, A. Y.; Pourghaderi, A.; Song, A.-Y.; Mouchaham, G.; Serre, C.; Reimer, J. A.; Bardow, A.; Smit, B.; Garcia, S. A holistic platform for accelerating sorbent- based carbon capture. *Nature* **2024**, DOI: 10.1038/s41586-024-07683-8.
